# Effects of renal pelvic high-pressure perfusion on nephrons in a porcine pyonephrosis model

**DOI:** 10.3892/etm.2013.1023

**Published:** 2013-03-21

**Authors:** JIAN WANG, DA-QING ZHOU, MENG HE, WEN-GANG LI, XIANG PANG, XIAO-XIANG YU, BO JIANG

**Affiliations:** Department of Urology, 303rd Hospital of People’s Liberation Army, Nanning, Guangxi 530021, P.R. China

**Keywords:** infection, nephron, renal pelvic pressure

## Abstract

The aim of this study was to investigate the effects of various renal pelvic pressure gradients on nephrons with purulent infection. Five miniature test pigs were selected. One side of the kidney was used to prepare the pyonephrosis model and the other side was used as the healthy control. A piezometer and a water fill tube were inserted into the renal pelvis through the ureter. Prior to perfusion, punctures were made on the healthy and purulent sides of the kidneys to obtain tissues (as controls). Subsequently, a puncture biopsy was conducted on the kidneys at five pressure levels: 10, 20, 30, 40 and 50 mmHg. Once the renal pelvic pressure had increased, the healthy and injured kidneys presented pathological changes, including dilation of the renal tubule and capsule and compression of the renal glomerulus. When the renal pelvic pressure exceeded 20 mmHg, the injured kidney presented more damage. Electron microscopy revealed that the increase in pressure resulted in the following: the podocyte gap widened, the epithelial cells of the renal capsule separated from the basement membrane, the basement membrane thickness became uneven, the continuity of the basement membrane was interrupted at multiple positions and the renal tubule microvillus arrangement became disorganised. The manifestations in the pyonephrosis model were more distinct compared with those in the healthy kidney. As the renal pelvic pressure exceeds 20 mmHg under a renal purulent infection status, the nephrons become damaged. The extent of the damage is aggravated as the pressure is increased.

## Introduction

As percutaneous nephrolithotomy continues to advance, the majority of upper urinary calculi do not require open surgical treatments. Percutaneous nephrolithotomy has become the main method of upper urinary calculi treatment, particularly for Staghorn calculi (1,2). However, percutaneous nephrolithotomy has its disadvantages. It requires persistent washing to maintain a clear field of vision during surgery. This procedure causes the washing liquid to be absorbed and the renal pelvic pressure to increase, thereby resulting in a backflow problem (3,4). When the kidney is injured or in a purulent infection status, the renal pelvic pressure increases, which causes backflow of bacteria into the blood, causing septicaemia of urinary origin and a higher mortality rate (5–8). Therefore, when the kidney is infected, the majority of medical doctors do not conduct one-stage percutaneous nephrolithotomy. Instead, they initially conduct percutaneous nephrostomy, followed by two-stage percutaneous nephrolithotomy once the disease condition has become stable (9–11). However, under certain conditions, conducting one-stage percutaneous nephrolithotomy is also feasible, even if the kidney has purulent infection (12). Thus, several questions arise, particularly concerning the procedures involved in conducting surgery when renal infection is present, as well as on the correlation between renal pelvic pressure and renal backflow. Currently, studies on these issues are rare. Therefore, investigating the correlation between renal pelvic pressure and renal backflow during renal infection is important. This study presents a pathological biopsy by preparing the porcine pyonephrosis model and implementing various perfusion pressures to observe the pathological changes in the kidney under different pressures and to investigate the effects of pressure on the infected kidney. This study also aims to provide information concerning the clinical surgical treatment of calculus pyonephrosis.

## Materials and methods

### Animals

Five miniature common male test pigs aged 2 months with body weights ranging from 25 to 30 kg were selected. They were fed conventionally. This study was performed in strict accordance with the recommendations in the Guide for the Care and Use of Laboratory Animals of the National Institutes of Health. The animal use protocol was reviewed and approved by the Institutional Animal Care and Use Committee (IACUC) of the 303rd Hospital of the Chinese People’s Liberation Army.

### Methods

Five miniature test pigs were used to prepare the pyonephrosis model. After the pigs had been adaptively fed for 5 days, the experiment was conducted. Prior to surgery, 10 mg/kg ketamine hydrochloride was administered by intra-muscular injection for anaesthesia. Next, 5 mg/kg ketamine hydrochloride was administered by intravenous injection for maintenance. Once the pigs were anaesthetised, they were laid on the left side while surgery was performed on the right ureter. Each pig was coeliotomised using a right waist incision to expose the right kidney and right ureter. At 5 cm from the right kidney, 2 ml supernatant of pig manure and normal saline was injected into the right ureter. When the injection had been administered, the abdominal cavity was closed. Following surgery, the pigs were fed normally and no antibiotic was administered.

After 3 days, the miniature test pigs were anaesthetised using the aforementioned method. The pigs were coeliotomised to expose the bilateral kidneys and ureters. Bilateral ureters were ligated 5 cm from the renal pelvis and a piezometer and a water fill tube were inserted from the ligation-proximal ureter towards the renal pelvis. At this time, the water fill tube was opened. The model was qualified after purulent urine was drained from the right injured kidney and clear urine was drained from the left healthy kidney.

Prior to perfusion, punctures were performed on the healthy and purulent sides of the kidneys to obtain tissues (as controls). Normal saline was perfused into the renal pelvis to allow the renal pelvic pressure to increase rapidly to 10 mmHg; this pressure was maintained for 10 min. Subsequently, the healthy and injured kidneys were punctured using one needle to conduct biopsy. Next, the perfusion liquid was drained and normal saline was perfused again to increase the pressure to 20 mmHg. When this pressure had been maintained for 10 min, the kidneys were punctured again. Similarly, puncture biopsy was performed at renal pelvic pressures of 10, 20, 30, 40 and 50 mmHg. The obtained tissues were restored in 10% formalin and electron microscopy fixation solutions.

### Optical microscopy

Haematoxylin and eosin (H&E) staining of a specimen was conducted. Afterwards, the morphological structures of the renal capsule, renal glomerulus and renal tubule were observed under an optical microscope. At the same time, images were captured.

### Electron microscopy

A specimen was cut into ultra-thin sections of 60–68 nm. A JEM-1010 model transmission electron microscope was used to observe the ultra-microstructures of the renal capsule, renal glomerulus and renal tubule. At the same time, images were captured.

## Results

### Pathological examination

**Figure 1:**
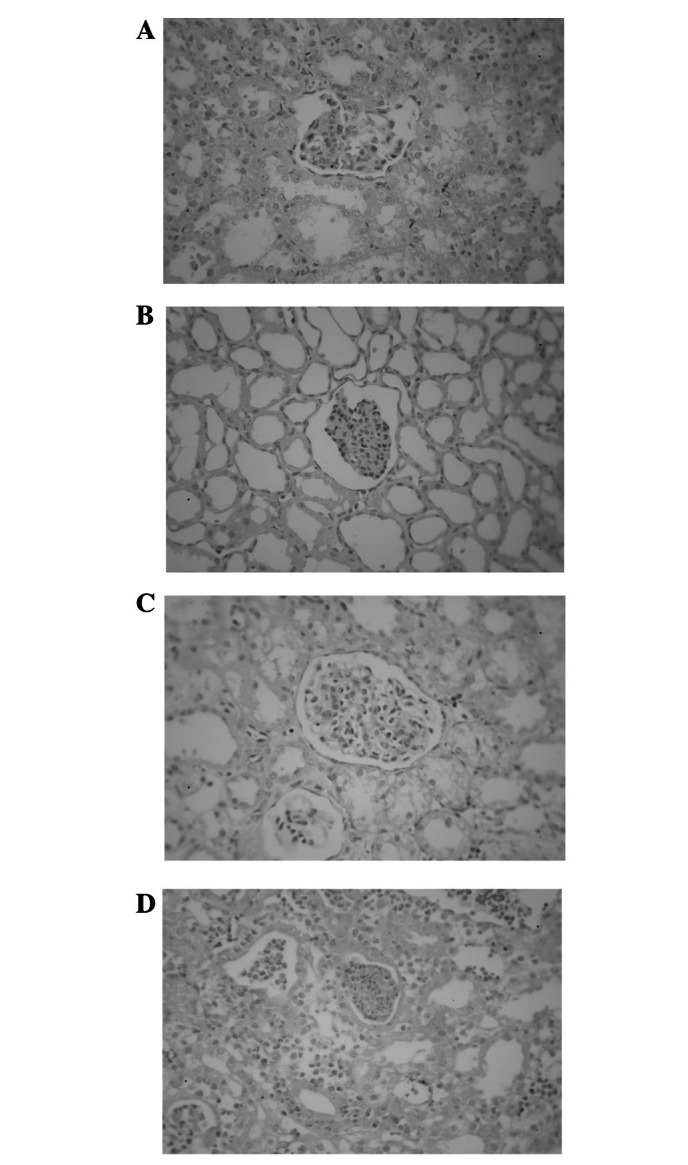
Pathological examination results. (A) When the pressure in the healthy side increased to 20 mmHg, the renal tubule dilated (H&E staining; optical microscope magnification, ×200). (B) When the pressure in the healthy side increased to 40 mmHg, the renal tubule and renal capsule were clearly dilated and the nephron structure was complete (H&E staining, magnification, ×200). (C) When the pressure in the infected side increased to 20 mmHg, the renal tubule and renal capsule were dilated and the continuity of the renal tubule basement membrane was partially interrupted (H&E staining, magnification, ×200). (D) When the pressure in the infected side increased to 40 mmHg, the renal tubule and renal capsule were clearly dilated and the continuities of the renal capsule and renal tubule were interrupted (H&E staining, magnification, ×200). H&E, haematoxylin and eosin.

When the renal pelvic pressure increased, healthy and injured kidneys presented pathological changes, including renal tubule and renal capsule dilations and renal glomerulus compression. At the same time, the purulent infected kidney exhibited interstitial oedema and inflammatory cell infiltration. When the perfusion pressure exceeded 20 mmHg, the purulent infected kidney exhibited degenerative necrosis of the renal tubule. When the perfusion pressure was increased to 40 mmHg, damage in the renal glomerulus and renal tubule became more evident and local structures of the renal glomerulus and renal tubule were damaged and destroyed. Under the same pressure, the healthy kidney exhibited dilations of the renal tubule and capsule and compression of the renal glomerulus. Less damage and fewer changes were observed in the healthy kidney (Fig. 1).

### Electron microscopy

**Figure 2:**
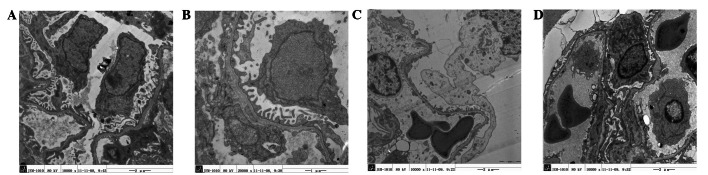
Electron microscopy results. (A) When the pressure in the healthy side increased to 20 mmHg, the separation of podocytes from the basement membrane was increased (transmission electron microscope; magnification, ×10,000). (B) When the pressure in the healthy side increased to 40 mmHg, the separation of podocytes from the basement membrane continued to increase and morphological disorders appeared (transmission electron microscope; magnification, ×10,000). (C) When the pressure in the injured side increased to 20 mmHg, the podocytes protruded and were clearly separated from the basement membrane, protuberances were flattened and the structure became disordered. Additionally, podocyte protuberance fragments were visible (transmission electron microscope; magnification, ×10,000). (D) When the pressure in the infected side increased to 40 mmHg, morphological changes of podocyte protuberances were acutely aggravated. Additionally, erythrocyte leakage occurred and leukocytes appeared (transmission electron microscope; magnification, ×10,000).

Different results were observed as the pressure was increased. The podocyte gap widened and the epithelial cells in the renal capsule separated from the basement membrane. The basement membrane thickness became uneven and its continuity was interrupted at multiple positions. The renal tubule microvillus arrangement became disorganised. The manifestations in the pyonephrosis model were more evident compared with those in the healthy kidney. In the renal capsule, inflammatory cells and transudatory erythrocytes were visible. In addition, microvillus disorder and shedding, mitochondrial vacuolisation, reduction in mitochondrial cristae and lysosomal expansion were visible at multiple sites (Fig. 2).

## Discussion

Upper urinary calculus is a common disease that causes kidney obstruction and hydrops. In physiological status, the renal pelvic pressure is ∼0.98 kPa (7.35 mmHg). In the early stage of kidney obstruction, renal blood flow perfusion increases and the renal pelvic pressure gradually increases until it reaches a maximum (8.82 kPa) (13). Following this, the renal blood flow begins to decrease, the renal glomerulus filtration rate consequently drops and the renal pelvic pressure gradually decreases, at times becoming reduced to the baseline or to a level slightly above the normal baseline. Furthermore, ureter dilatation and renal pathological changes occur (13). Therefore, a basic method of relieving renal obstructive lesions is the prompt removal of the obstruction. Percutaneous nephrolithotomy is a common treatment method for the removal of calculus obstruction. However, it requires persistent perfusion and washing during surgery. Thus, renal pelvic pressure is likely to increase rapidly within a short time, which easily affects the renal structure and function. The effect is most evident in cases of complicated purulent infection.

According to the results of an *in vitro* study (14), a renal pelvic pressure >35 mmHg causes persistent reverse flow in the renal pelvic veins and lymphatic vessels. In cases of infection, a renal pelvic pressure of 15–18 mmHg may cause reverse flow. According to Kukreja *et al*(15), perfusion liquid absorption occurs during percutaneous nephrolithotomy. Even when renal pelvic pressure is <30 mmHg, perfusion liquid absorption continues to occur. During high-pressure perfusion, the perfusion liquid is absorbed through the impaired collection system mucosae and the open vessel of the puncture channel and the maximum amount of reabsorbed liquid may reach 474 ml. Furthermore, post-operative fever, pain and reductions in haemoglobin levels are all related to perfusion liquid absorption. For certain patients with complications involving heart and lung diseases and renal inadequacy, or even children, large amounts of absorbed liquids cause body fluid levels to increase excessively (16). If the calculus is complicated with bacterial infection, the absorbed bacteria cause bacteraemia. Therefore, administering furosemide following surgery is helpful in post-operative recovery. Using the radioactive labelling method, Stenberg *et al*(17) identified that liquid backflow sites are located in the renal calyceal fornix region. The liquid also undergoes backflow through the renal veins, instead of the lymphatic system. *In vitro* renal model studies (18) have shown that high pressure in the renal pelvis is related to the size and length of the puncture sheath and its position in the kidney. Therefore, decreasing the renal pelvic pressure during surgery is necessary to expand the puncture channel or decrease the height of the perfusion liquid (19). Ng *et al*(20) conducted micro-channel percutaneous nephrolithotomy on patients in a supine position. When the working sheath was placed at a position lower than the renal collecting system, high-pressure perfusion liquid flowed from the gap between the working sheath and the percutaneous nephroscope due to gravity and the renal pelvic pressure was significantly less than that when the patient was in a prone position. Furthermore, effective intra-operative gravel washing was conducted and the post-operative complications were slightly reduced. These observations indicate that perfusion liquid absorption or backflow caused by an increase in the renal pelvic pressure is an important factor that causes various post-operative complications. Minimising the corresponding complications by decreasing the intra-operative renal pelvic pressure also becomes possible.

The current study investigated the effects of renal pelvic pressure on the kidney. The study was specifically designed to investigate the correlation between nephron damage and renal pelvic pressure with renal purulent infection. Since a porcine kidney is extremely similar to a human kidney, the porcine renal model efficiently represents the progressive situation of the same lesions in humans. This study shows that an increase in the renal pelvic pressure within a certain range (<50 mmHg) for a healthy kidney causes renal tubule and renal capsule dilation and renal glomerulus compression. Less damage and fewer changes were also observed. For a kidney with purulent infection, renal tubule degenerative necrosis was observed at a perfusion pressure >20 mmHg. When the pressure increased to 40 mmHg, damage in the renal glomerulus and renal tubule became more evident and local structures in the renal glomerulus and renal tubule were damaged and destroyed. The same changes were visible under an electron microscope. When the renal pelvic perfusion pressure was increased to 20 mmHg, the thickness of the renal glomerulus and renal tubule basement membrane became uneven and the continuity of the basement membrane was interrupted at multiple positions. The experimental results demonstrate that the pressure resistance of a kidney with purulent infection is significantly lower than that of the normal kidney. If treatment is conducted at this time, pathogenic bacteria invade the vessels at the damaged basement membrane and cause bacteraemia or septicaemia through the blood circulation. Therefore, when percutaneous nephrolithotomy is performed for renal calculus complicated with infection, strictly control of the intra-operative renal pelvic pressure is necessary. Maintaining the pressure below 20 mmHg is also relatively safe. This study investigated only the effects of renal pelvic pressure on a kidney with acute infection. However, chronic renal infection is more common in clinical practice. Therefore, further studies on the effect of chronic renal infection are required to determine whether our results would be consistent with those of acute infection.
